# From the “metaphysics of the individual” to the critique of society: on the practical significance of Michel Henry’s phenomenology of life

**DOI:** 10.1007/s11007-012-9226-9

**Published:** 2012-08-08

**Authors:** Michael Staudigl

**Affiliations:** Department of Philosophy, University of Vienna, Vienna, Austria

**Keywords:** Material phenomenology, Michel Henry, Theological turn, Transcendendal corporeality

## Abstract

This essay explores the practical significance of Michel Henry’s “material phenomenology.” Commencing with an exposition of his most basic philosophical intuition, i.e., his insight that transcendental affectivity is the primordial mode of revelation of our selfhood, the essay then brings to light how this intuition also establishes our relation to both the world and others. Animated by a radical form of the phenomenological reduction, Henry’s material phenomenology brackets the exterior world in a bid to reach the concrete interior transcendental experience at the base of all exteriority. The essay argues that this “counter reduction,” designed as a practical orientation to the world, suspends all traditional parameters of onto(theo)logical individuation in order to rethink subjectivity in terms of its transcendental corporeality, i.e., in terms of the invisible display of “affective flesh.” The development of this “metaphysics of the individual” anchors his “practical philosophy” as he developed it—under shifting accents—throughout his oeuvre. In particular, the essay brings into focus Henry’s reflections on modernity, the industry of mass culture and their “barbaric” movements. The essay briefly puts these cultural and political areas of Henry’s of thinking into contact with his late “theological turn,” i.e., his Christological account of Life and the (inter)subjective self-realization to which it gives rise.

Men debased, humiliated, despised and despising themselves, trained in school to despise themselves, to count for nothing—just particles and molecules; admiring everything lesser than themselves and execrating everything that is greater than themselves. […] Men turned away from Life’s Truth, caught in all the traps and marvels where this life is denied, ridiculed, mimicked, simulated—absent. Men given over to the insensible, become themselves insensible, whose eyes are as empty as a fish’s. Dazed men, devoted to specters and spectacles that always expose their own invalidity and bankruptcy […] *Men* will want to die – but not *Life*.^1^

Paul Ricœur’s oft-cited dictum that the history of phenomenology is a “history of heresies,”^2^ seems to apply in an extraordinary manner to the philosophy of Michel Henry. While he takes up and furthers phenomenology, he nevertheless shapes it in a way that is not only particular and critical but also quite unfamiliar. As Waldenfels has established in his well-known work on *Phänomenologie in Frankreich*, Henry’s philosophical approach stands out for the fact that he is beholden to few of the intuitions essential to phenomenology. That said, he ultimately pursues one that possesses enormous explosive force, namely, the insight that affectivity is the most primordial mode of revelation of both our self and the world.^3^ In the following, I would like to demonstrate the explosive force this intuition takes on when it is applied, as Henry himself has indeed done in diverse respects, to the problems of practical philosophy and in particular the critique of culture and questions of political philosophy. For I believe that the significance of Henry’s thought in no way belongs solely to the theoretical register. On the contrary, it seems to me that the force of his thought is principally discovered in its practical unfolding and application.

For Henry, in contrast to Husserl and classical phenomenology, what is at stake is no longer the question of how to think life on the basis of the world—or on the basis of another transcendental horizon such as time, the body, or the other—but rather, on the contrary, it is a question of how to think the immanent capacity of revelation of life itself and therefore of how to think the world on the basis of life. Perhaps the most difficult aporia in his thought consists precisely in what it means to *think life*.

Given such a definition of the task of phenomenology, a radical paradigm shift from a “phenomenology of transcendence” to a “phenomenology of immanence” is announced and thus a shift from intentionality to praxis. Hence the task of phenomenology, rightly understood, consists in working out the immanent and, for Henry, practical *intelligibility of life* as the principle or *logos* of appearance per se—i.e., of all phenomenalization.^4^ One is inclined to ask, however, which phenomenology? For if phenomenology—as we see in the thematization of intentionality in Husserl or the transcendence *qua* essence of the appearing of what appears in Heidegger—in fact already inquires into nothing other than the logos of appearance, then will it not get it lost in Henry’s conception of it in a “tautological interiority” or “immanent autoreference” of life?^5^ We should ask what is meant by *life* here, especially when “absolute life” is placed in scare quotes or when it capitalized and when it is affirmed, “there is but a single and selfsame Life” and that it has “*the same meaning for God, for Christ, and for man*”.^6^ Do not such declarations signify a surrender of phenomenology to (crypto-)theology, suggested, along with others, by Dominique Janicaud, who counts Henry in his polemical essay on the “theological turn” among the “new theologians”?^7^ In order to adequately counter this all too sweeping charge and ultimately to be able to develop the important implications of Henry’s position for a thinking of culture and politics, we should envision what is at play in his transformation of phenomenology into a “phenomenology of life”: it concerns, to foreshadow this crucial point of his discourse, the *transcendental humanitas* of human beings, our inescapable destiny to be a *Self*, something that is itself given in its “living flesh”^8^ without owing its singularity to any kind of transcendent principle, but that always finds itself exposed to the seductions of transcendence, that is, by the attempt to define itself by means of a transcendent principle. That and how such an unfathomable singularity is at play in our cultural and political self-understanding is shown in Michel Henry’s practical writings.^9^

## Turning phenomenology on its head: from intentional to “material” phenomenology

For Henry, the common presupposition that links classical philosophy with historical phenomenology—from Husserl and Heidegger to Merleau-Ponty and Levinas—consists in the fact that they think the logos of phenomena as the logos of the world. To put it another way, they all think appearing as horizontal appearing. For this reason, Henry believes, they are not capable of explaining how intentionality brings itself forth, that is, how transcendence is able to transcend itself.^10^ In order to solve this problem it is necessary to go back to the immemorial ground of experience, in which, as Henry puts it, intentionality takes possession of itself, or, rather, “experiences itself” (*s’éprouve soi*-*meme*). Only in such a way of primarily giving itself to itself is it also capable of transcending itself and moving towards the other. For Henry, the task of a radical, i.e., “material phenomenology” thus consists in returning to “pure immanence” and its inner “structure” and “dynamic.” Against the “ontological monism”^11^ of Western philosophy, i.e., the presupposition that “phenomenological distance is the ontological power which gives us access to things,”^12^ such a phenomenology thematizes the “duplicity of appearing.”^13^ This implies that against manifestation—which is Henry’s term for all transcendent, ecstatic, or worldly appearing—is pitted a pure appearing or, to be more precise, a self-appearing or auto-revelation. The concept of duplicity underscores the fact that this distinction is no way meant as a dichotomous comparison, but rather that what should be thought is a foundational relationship: ultimately, Henry postulates, the essence of manifestation is founded in nothing other than precisely this self-appearing. This does not takes place, however, within thought, representation or reflection, but rather in the mode of affectivity, or, stated more precisely, in the mode of *auto*-*affection* on the part of pure phenomenological life.

For Henry the decisive presupposition of classical phenomenology thus lies in the fact that the life of consciousness is to be realized within the horizon of exteriority, visibility, or simply the world, i.e., in the domain of a living subjectivity that in-tentionally exceeds itself. In such exteriority it is through intentionality that the subject does not coincide with itself inasmuch as it always differs from itself, fractured by the difference of the world itself. In Henry’s view it was Husserl who first contemplated this insight about intentionality, which has been radicalized by post-Husserl phenomenology, and has also influenced deconstruction. However, when it is a question of thinking the proper essence of selfhood, Henry takes all of these positions to be entirely insufficient. According to him, Husserl’s theory of “self-constitution” puts us on an aporetic path. It does this by privileging an intentionality that constitutes objects within its temporal flow at the expense of a more primal self-presencing prior to the streaming of time.^14^ The question that arises here about a primordial selfhood had always remained a problem for Husserl and classical phenomenology (and, of course, it was in no way only a phenomenological problem). According to Henry, the task of rendering intelligible this primal self-presencing in its passive/affective foundation was an impossible one for Husserl to achieve because he emphasized the cognitive structure of consciousness and its various intentional faculties (e.g., presentation, representation, imagination).^15^

Henry goes back to Husserl, however, in order to pose this question in a manner that is adequate to the task. Central for him is an early insight suggested by Husserl in his lecture, *The Idea of Phenomenology*, one that is quickly forgotten and never developed in his subsequent writings. Husserl’s insight is that nothing other than pure appearance as such, hence phenomenality, is the basic theme of phenomenology, and therefore not the primacy of the phenomenological ‘gaze’ (*Schau*), nor, more generally speaking, that of theory.^16^ Phenomenality here means nothing other than the condition under which something in general is initially capable of attaining the status of a phenomenon. We must add, however, that we can no longer appeal to a horizon, in which the staging of the “thing itself” would unfold (i.e., objectivity in Husserl, Being in Heidegger, or the “flesh of the world” in late Merleau-Ponty, etc.). Rather, if one considers appearing in the dimension of its origin, particularly in regards to such a horizon, a problem arises, namely, that as a method phenomenology immediately loses sight of this pure appearing. This is, one might say, its fate, insofar as it takes itself to be a process of making evident, as a mode of making-visible (*Sehen*-*lassen*), ex-plication or a mode of rendering-manifest. To put it in connection with traditional concepts: Henry argues that intentionality, by virtue of its constitutive character whereby it sets phenomena within a horizon, cannot initiate us into the proper domain of phenomenological inquiry. Intentional consciousness—i.e., that which bears the distinction of being conscious of something as something—is thus not primordially responsible for making the world open to us. As a method of beholding and grasping (*Schauen und Fassen*), intentional phenomenology in fact conceals the genuine phenomenological register that Henry has in mind. Thus, in contrast to Husserl, who orders the original relation to the world by way of intentional reflection or “consciousness of…,” Henry does not. This is to say that Husserl insists that intentional life relates the self to something different than itself. And it is through this relation that the true and only possible “access to being” is possible, which renders intentionality, as Fink aptly puts it, the central hypothesis of Husserl’s phenomenology.^17^

This Husserlian hypothesis, which posits consciousness, understood as originarily intentional, as the genuine mode of accessing Being yields an extraordinary phenomenological situation: if the primordial “How of givenness” or of the “thing itself” is identified with intentional display, then the appearing of the being is substituted for the appearing as such:

Still expressed in a language that demonstrates the close affinity between this historical phenomenology and classical philosophy, every consciousness is a ‘consciousness of something’. Thus we have, on the one hand, the appearing (consciousness) and, on the other hand, the something, the being. In itself the being is foreign to appearing and thus unable to phenomenalize itself through itself. For its part, the appearing is such that it is necessarily the appearance of something other, of the being. Appearing turns away from itself in such a radical and violent way that it is directed entirely to something other than itself, namely, the outside—it is intentionality. Because appearing *qua* intentionality finds itself thus essentially displaced in what it allows to appear, appearing no longer appears, but only that which appearing lets appear within itself: the being. As a result, the object of phenomenology, the ‘thing itself’, is distorted in such a way that the object of phenomenology is no longer the appearing but rather the appearing of the being and ultimately the being itself, insofar as it appears.^18^

In contrast, what Henry provides is the “re-inscription of intentionality in the non-intentional.” He determines the non-intentional as a ground that is “older” than intentionality. Henry puts into play a radical reduction that reduces or disqualifies the world as the horizon of intentional exhibition and site of any and all givenness. Horizon-inscription and reduction to the ego are hereby suspended as constituents of the phenomenon’s genesis. In regards to this methodological approach Henry speaks of a “counter-reduction.”^19^ This brings us back to the passive and affective foundation of the self, its pure self-appearing or its pure self-revelation, which are carried out in the “night of subjectivity,” in the “invisible” life of our affectivity.

The path from a “phenomenology of the world” to a “phenomenology of life,” from a phenomenology of “transcendence” to one of “immanence” has now been sketched more clearly. The duplicity of appearing implied here is central for Henry’s thought. He holds on to this duplicity against the “ontological monism” that reduces the field of appearing entirely to the laws of intentional display that exhibits objects in the luminous horizon of the world. Nevertheless, for him it is not a question of construing this duality or, rather, duplicity, as a *dualism*.^20^ His aim lies rather in grasping the world on the basis of life, without thinking the latter—*qua* nature of the subject *and* of Being—within the “apperceptive horizon” of the world (nor therefore in that of Being, but also not in that of alterity or a mysterious “flesh of the world,” etc.), but rather purely from out of its “immanent teleology.”^21^ We find this idea already in the definition Henry gives of the self in *L’essence de la manifestation*:

That which is thus forever burdened with self, only this can we truly call a Self. Herein is accomplished the movement without movement in which it receives, as a substantial and burdensome content, that which it is; it masters itself, arrives at itself, experiences its own profusion. *The Self is the surpassing of the Self as identical to self*.^22^

This “ipseization” of the subject—as it is called in *I am the Truth*—takes place in its living bodily praxis, i.e., purely in the invisible medium of its affectivity as well as practically lived intelligibility. This suggests that we speak here of a *metaphysics of the individual*, since for this reason all traditional parameters of onto(theo)logical individuation—such as substantiality, spatio-temporality localization, self-determination, self-motivated agency (*Selbsttätigkeit*) or spontaneous self-determination—are suspended—and this in favor of the apodicticity of an unshakeable foundation of our practical “knowledge of life” (*savoir de la vie*) as a purely individuated self-feeling.^23^

Against this background, Henry’s central thesis can be elucidated, namely, that the mode of the self-appearing or the self-revelation of life is not the affection by means of something foreign to consciousness, nor external (as classical philosophy would have it), nor is it the affection by means of itself as something other in the medium of time or the faculty of imagination (as Husserl and Heidegger would have it), but rather the pure auto-affection of life, which is revealed in the feeling it has of itself. Thus, Henry considers the affective auto-revelation as the ineluctable condition that precedes all intentional relatedness and “being-toward-the-world” in general. For instance, the “impressional content of the world” is no longer that which fills the “meaning-bestowing” intentional ray, but rather—and thus runs Henry’s thesis—to a non-intentional auto-affection of life as a pathos interior to the ego. According to Henry this pathetic, i.e., radically passive, self-appearing constitutes the *invisible* reality of life in its self-affection.

What Henry designates as auto-affection, moreover, precedes the classical distinction between activity and passivity. It is more primordial than the passivity involved in the suffering of an external force and also the passivity of feelings, emotions, affects, and dispositions. Thus what is at issue is an originary, i.e., ontological passivity of affectivity. For Henry, this originary passivity is at once passive and yet constitutive of the ground of selfhood itself, and this represents one of the decisive thematics of his system. For this is implied in every feeling as its “self-feeling,” or originarily passive self-relationality.^24^ There is no difficulty in claiming that Henry’s theory of the irrepressible pathos of auto-affection is distinct from affective states like sensation, which exhibit an intentional or exterior relation to the world. So exterior sensations involve a relation to something different than themselves, (to outside sensory impressions), an affection structured by what is known as hetero-affection. For Henry, hetero-affection, while a legitimate field of display, does not exhaust what affectivity is. Or, to express it another way, “Henry is convinced that the events of the world are only occasional causes of our feelings. It is our affectivity itself which makes it possible for the world to exert an influence upon our interior life. In other words, it is, in each case, our particular ‘attunement’ that exposes us to an affection by the world.”^25^ In other words, not what befalls us from outside determines affectivity but rather affectivity in its self-relation interior to itself first makes possible any experience of what might befall us. That all dispositions, feelings, etc. here are given to themselves without the possibility of recoil in their “being-always-already-given-to-themselves,” “crucified” on themselves, as Jad Hatem puts it,^26^ implies that they bear the character of *suffering*. That said it is not the case that life only *suffers* in such feelings and dispositions. Henry understands suffering rather as a “path”. Inasmuch as it surpasses itself to “return” home to itself, this “being-itself,” its self-givenness, is also a fount of pleasure and joy that never runs dry. Power and impotence are thus intertwined in this interior experience of a “gift which cannot be refused,”^27^ and so draws life from the auto-donation of life inside the ego as a “primordial force” or “drive,” both words deployed by Henry himself. This process of arriving at itself (*venue en soi*), this *gift* of life (*don de la vie*) that can never be refused, Henry understands as the gift of *absolute* life, which, traditionally speaking, we would call God. The “transcendental birth” of the living (*le vivant*) in (absolute) Life therefore in no way describes a factual genesis or individuation, but rather a *conditio*: it concerns the *conditio* *of sonship*, as Henry notes in his Christological transformation of his major phenomenological intuition in *I am the Truth*:

To understand man on the basis of Christ, who is himself understood on the basis of God, in turn rests on the crucial intuition of a radical phenomenology of life, which is precisely that of Christianity: namely, that *Life has the same meaning for God, for Christ*, *and for man*. This is so because there is but a single and selfsame essence of life, and, more radically, a single and selfsame life. This life—that self-generates itself in God and that, in its self-generation, generates the transcendental Arch-son as the essential Ipseity in which this self-generation comes about—is the Life from which man himself takes his transcendental birth, precisely since he is Life and is explicitly defined as such within Christianity. He is the Son of this unique and absolute Life, and thus the Son of God.^28^

This Trinitarian conception of the self-engendering of absolute life, and of the generation that takes place within the Trinity of a “first living being” (i.e., Christ), through which generation this absolute Life realizes itself, and finally this conception of the generation of each living being (individual) in and through this Life, reaches a Christological apex: the “first living being” born in perfect reciprocity with absolute Life Christ represents for Henry that primordial or “older ipseity” of a “First Self”^29^ through which alone each living being can obtain the unrefusable gift of life.^30^

Henry’s turn to Christology as a way to explicate life’s living relation to humanity should not be dismissed as a theological domestication of phenomenology. Viewed phenomenologically, we see that he forges a noteworthy modification of the concept of self-affection. Henry seems to be responding to the critical charge made against his theory of auto-affection, namely, that pure immanence possesses no structure or history and therefore it excludes every possible relation to otherness, as well as every source of hetero-affection.^31^ On the contrary, Henry introduces the distinction between a strong, absolute, or naturalizing self-affection, on the one hand, and a weak, relative, or naturalized self-affection, on the other hand.

The singular Self that I am experiences itself only within the movement by which Life is cast into itself and enjoys itself in the eternal process of its absolute self-affecting. The singular Self self-affects itself; it is the identity between the affecting and the affected, but it has not itself laid down this identity. *The Self self*-*affects itself only inasmuch as absolute Life is self*-*affected in this Self.* It is Life, in its self-giving, which gives the Self to itself. It is Life, in its self-revelation, that reveals the Self to itself.^32^

Although we cannot discuss the Christological transformation of Henry’s thought in more detail here, the following point seems to have a particularly salient significance for our context: according to Henry, the experience that life has of itself takes place in connection with the aforementioned pathic duality of suffering and enjoyment, i.e., the “antinomic structure of life”.^33^ Inasmuch as this “antinomy of the fundamental affective tonalities”^34^ of Suffering and Joy is “co-constitutive of life’s self-revelation” and thus “refers back to a primitive unity,” it constitutes selfhood, or, as Henry also puts it, *ipseity*. In other words, since human life is given to itself in its pure passivity, it is not self-positing or self-subsisting and thus not “the source” of its own condition as living—rather Henry insists that I “find myself” thrown into life, self-affected, as he articulates it in *I am the Truth.* 35 Ipseity is thus neither *absolute* self-affection, nor does it result from the *impersonal* execution of anonymous forces or drives.^36^ Ipseity so understood by Henry means having a *self* to the extent that life in its self-affection surpasses itself into “affective formations” and thus transforms itself by way of that affectivity, i.e., insofar as in the originary affectivity of feeling-itself it also experiences the essence of its subjectivity. Insofar as ipseity carries the burden of feeling-itself and, in its condition as son, experiences it as the burden of its own given being, it comes into “possession of itself and of each of the modalities of its life”^37^—*and, hence, becomes the subject that takes up a relation to the world*.

## On the crisis of the cultural reality

To shed light on the innovative significance of his practical philosophy, we must develop a deeper understanding of how Henry thinks the aforementioned *relation* to the world. It concerns—and this is crucial to note—a “world,” which would not be the *pre*-*given* aim that is exterior to the self-movement of life. For Henry, “world” is not to be understood in the traditional sense of “the totality of all things”, nor is it to be taken phenomenologically as the “horizon of horizons,” nor is it even the “medium of care.” With the term “flesh” (*chair*) Henry designates—in sharp contrast to late Merleau-Ponty who takes it to represent the common milieu of subject and object—an immanent arch-praxis, that is, the transcendental “I can” of the subject. In the phenomenality of this “I can” world-content and the execution of our potentialities are inextricably bound together at the basic level of our transcendental affectivity prior to the display of the world:

If the world is everywhere unrelenting, if the fabric of the sensible is perpetuated without fail or lacuna and it does not come unraveled at any point, if every fiber and every grain of which it is composed are interminably evocable, it is because every one of the capacities (*pouvoirs*) that lead me to them is the power of a flesh nothing is able to divide (*sépare de soi*), that is always present to itself in a memory without diversion, without thought, without a past, without memory—in its immemorial memory. It is my flesh that is indivisible (*indéchirable*). The unity of the world is thus an immanent unity, it lies in the parousia of my flesh.^38^

The concrete experience of the world thus “demands,” Henry concludes, “as its ultimate possibility, a consciousness without world, an acosmic flesh.”^39^ By this he understands, following Maine de Biran, the “immanent corporeality” of our “I can”. This “transcendental I can” is to be thought as a living ability given to us, a capacity that first and foremost makes possible the unlimited repetition of our concrete capacities.^40^ The task of unfolding the auto-affective structure of life thus is assigned to the flesh as the material concretion of the self-givenness of our innermost selfhood, i.e., ipseity. The flesh accomplishes, as it were, its translation into “affective formations” and therefore embodies “the fundamental *habitus* of transcendental life,”^41^ which make up the “life-world” as a *world of life* in its innermost essence.^42^

With this we have a further indication of how transcendence (i.e., the world) arising from immanence (i.e., life) is to be understood then as something other than a “non really included”^43^ transcendence (*Transzendenz irreellen* *Beschlossenseins*)—namely, as “affective formation”, “condensation”, or even as the “immemorial memory” of our flesh. Yet might these descriptions of life’s self-movement be represented more precisely? How are we to think Henry’s claim that “the world’s reality has nothing to do with its truth, with its way of showing, with the ‘outside’ of a horizon, with any objectivity”?—how are we to think that the “reality that constitutes the world’s content is life”?^44^ Viewed against this background, Henry’s theory of the duplicity of appearing ostensibly leads to a seemingly insurmountable problem: how can the notion of an “acosmic flesh” in its “radical independence”^45^ as the sole reality of life actually found that which is outside of it, the world? It is precisely this that we must now reflect on more explicitly if we wish to show that his approach can be made useful for problems that arise in the philosophy of society and culture as well as the questions posed by political philosophy. The main objection to Henry’s reinscription of the world within life proceeds in the following way: the “counter-reduction” aims to found the visible display of the world in the invisible self-revelation of absolute life, yet doesn’t this disqualification of the world set into operation a “complete scorn for all of life’s actual determinations”^46^ in the world? With this all too radical inquiry into the originary do we not become trapped in a “mysticism of immanence,” that remains enclosed in its own night, forever incapable of being expressed and coming into the world? To summarize Bernhard Waldenfels’ exemplary formulation of this critique, “doesn’t the negative characterization of self-affection as non-intentional, non-representational, and non-sighted or non-ecstatic bear a constant reference to the world-relation (*Weltbezug*) that it suspends?”^47^, ^48^

Henry would reply firmly in the negative to this question. On the contrary, he would posit that only a non-intentional phenomenology is capable of grounding the reality of the world, as a living reality that has nothing to do with its objective truth—i.e., with the truth as we express it *in* the world. This is certainly not meant to imply that our life brings forth the concrete content of the world, but rather simply that the lived intelligibility of such content is ontologically possible only as living content. In other words, its intelligibility rests on nothing other than the variation, transformation, and augmentation of those concrete individual potentialities that are at work in advance of its worldly appearing. According to Henry, Husserl was completely aware of this, however he failed to follow through on this insight:

Husserl’s phenomenology has not disregarded the non-intentional. On the contrary, it has designated the non-intentional *qua* *hylé* as a fundamental stratum of consciousness. […] Nevertheless this decisive function of the primary impression that would make of hyletic phenomenology the fundamental discipline of phenomenology becomes immediately overturned. The *hylé* is nothing more than matter for a form that alone has the power of illuminating it and of making of it a phenomenon, and this form that sheds light on it is intentionality. Thus the original essence of phenomenality as revelation of the impression *qua* impression, that is, its impressionality, or rather its affectivity, is concealed. […] With this concealment of the secret source and original essence of phenomenality at the hands of ecstatic phenomenality, it is nothing less than the concrete life of men that is carried away from the domain of philosophical thought, and away from that thinking which is opened up by phenomenology itself to exteriority and ultimately to a ruinous objectivism that is covered over by the objectivism of Galilean science—which was defined by its exclusion of subjectivity, that is, of life.^49^

Henry’s criticism of Husserl notwithstanding, this quote shows the extent to which his work is to be understood as a continuation of the Husserlian crisis-diagnosis.^50^ Indeed it should be noted that “objectivism” here should not be taken to mean only a reduction to the finality of the objective sciences inaugurated by Galileo (and the loss of their significance for life to which it necessarily leads), which Husserl reproaches for being a “weary” form of reason.^51^ “Objectivism” designates here rather the underlying self-conception of the human being as subject in light of “ontological monism,” given that such a self-conception still underlies the procedure of these sciences and is authorized anew by classical phenomenology. The human being, insofar as he or she is reduced to the subject of self-knowledge or to self-regarding care (*Sorge*), is subjected to a systematic elimination of his transcendental *humanitas*. It is therefore this transcendental *humanitas* that Henry names the field of invisible appearing that makes possible the ego’s praxis in the luminosity of the world. So understood, Henry does not eliminate the world as a field of appearing. He rather insists that there is a duplicity of appearing, i.e., both visible world and the invisible transcendental ipseity are qualified by its living auto-affection. It is only when the field of appearing is reduced to the world alone that phenomenology gives way to a barbaric character, eradicating the invisible life in which each ipseity takes hold of its life. Thus, in contrast to other analyses of barbarism, Henry does not speak in *La barbarie* of Auschwitz, the Gulag or other cases of genocide. The barbarism that he has in mind concerns rather a *self*-*destructive reduction of human knowledge*, which, according to him, made these twentieth-century catastrophes possible in the first place: *scientism*. By scientism Henry understands the view that “there exists no mode of knowing other than Galilean science, that is to say, modern physics.”^52^ Following the presupposition shared even by the humanities of a universal abstraction of all subjective qualities, we consequently find that our knowledge of the human being becomes circumscribed entirely within the display of the world, its exterior objectivity, its “truth.” In this context, Henry’s critique of culture is not merely concerned with the “forgetfulness of the life-world” nor the one-dimensionality of technology as framing (*Gestell*) and its resulting ethical problems, which dominate philosophical discourse today. These problems are thoroughly thematized in his recurring and decidedly conservative critique of culture.^53^ At its core, *Henry’s analysis aims at nothing other than the possibility of a destruction of life by means of itself, a possibility that underlies all these manifestations of crisis* and becomes more or less deliberately exploited by such crises.

Henry’s thesis implies that the germ of all barbarism lies in a self-destruction of life. The possibility that life may wish to destroy itself is no way obvious as already a cursory look at the philosophical tradition that extends from Spinoza through Hegel and up to Nietzsche and Heidegger shows. According to Henry, however, this possibility is indeed constitutive for life and is based on its duplicity. In order to understand this possibility it is necessary to examine the dynamic in which life in its affects is driven against itself, i.e., the irreducible arch-passive self-givenness of life. As to Henry, this passivity of life vis-à-vis itself, which, as we have said, is the germ of its augmentation and therefore the condition of any kind of culture, can lead life to turn against itself. Under what conditions, however, is this possible? Under the condition that it becomes no longer capable in itself to be augmented, under the condition that it is not adequate to the task of implementing the ground of its being, for such implementation belongs to its essence:

The assertion that life experiences itself (*s’éprouve soi*-*même*) implies that it is passive vis-à-vis itself and consequently vis-à-vis a foundation that sustains it […] Thence, there is room for a meditation or an effort that seeks to feel this foundation of Being within us more lively.^54^

Religion, mysticism, art, ethics, and rational knowledge but also the refined fulfillment of our most basic needs are exemplary cases in the realm of culture of the self-augmenting articulation of the “excessive” nature of life, i.e., its irreducibly antinomic nature. Thus, what is at play for Henry in these “symbolic institutions”^55^ is in no way merely the pre-scientific validity of subjective experience in contrast to the objectivism of the scientific worldview. At play rather is the innermost essence of this experience itself *qua* “cultural life-praxis” that expresses the excessive nature of life without “displacing life in what is outside life.”^56^ Henry's critique of traditional philosophical accounts of culture thus focuses on their tendency to regard culture as a means to expend or objectify life's energy in worldly products. Hence, Henry's task is not, as in Husserl, to unveil the “history of institutions”^57^ (*Stiftungsgeschichte*) that concretely articulates the symbolic matrices of our various socio-cultural life-worlds.^58^ For Henry it is rather a matter of inquiring into the *originary passivity of life as that which in its very pathetic self*-*movement oscillates between the self*-*delighting capacity for living and the self*-*agonizing wish to live no longer, but which will never be able to escape from this movement*. On these premises it can be established that what scientism or the “scientific knowing” attempts to flee when it excludes the concrete field of invisible display internal to the ego, or what we attempt to flee when we pretend to live out our subjective potentialities by appealing to “ideal entities,” is the self-experiencing (*épreuve de soi*) that necessarily involves the self-agonizing aspect of life.^59^

This flight from the invisible sphere of life into the exterior world of history and temporality however effectively takes on monstrous forms when life strives to sever itself from itself in order to bring this flight to its injurious yet unreachable end:

The leap outside of the self is a flight into exteriority in which it is a matter of fleeing oneself and thus of ridding oneself of what one is, that is, of the burden (*poids*) of this malaise and suffering. However, this flight remains caught in its own pathos. Thus there remains only one way out: in order to destroy purely and simply this malaise and this suffering of which we cannot be rid, which indeed have their possibility in our self-experience (*le s’éprouve soi*-*même*) and thus in life, life itself, its proper essence, must be terminated. This self-destruction is bound to be just as unsuccessful in its aim (*fins*) as is self-flight if it is true that the act of self-destruction is only possible on the condition that it actualizes and affirms the essence that it wishes to annihilate.^60^

Life preserves itself even in its intention to destroy itself. Barbarism, says Henry, is an “idle energy”,^61^ an energy that no longer traverses the suffering proper to it for the sake of augmenting itself. Out of this unbearable situation of life, which in its attempt to destroy itself cannot leave itself behind, results a fury of “self-flight.”

The flight of the self is the title under which almost everything that passes before our eyes is arranged. Not science in itself, which as knowledge of the nature that it defines in its procedures is completely positive but […] the belief that this Galilean science of nature constitutes the sole possible way of knowing, the sole truth, so that there is no other reality, as true reality, i.e., real reality, than the object of this science, such that man himself is not real except under this title and such that all knowledge that concerns him can only be a mode or form of this particular science. An ideology—scientism and positivism—has here become substituted for science.^62^

In no way does self-flight take place merely at the theoretical level. In the wake of this self-flight, which sets into motion scientism’s tendency to systematically put life out of play, we see a proliferation of barbaric practices, in which life is “expelled from itself.”^63^ Henry identifies several such practices, from the depreciation of life in social engineering, scientific practice and the tele-technological imaging and simulation of life to the destruction of the university. What is common to these practices is that they create an “inhuman world”^64^—that is, a world that is “perpetually external”—whose truth disposes of the actuality of the life that makes it possible. In such practices, life is subjected to a fundamental *ontological shock*, for it becomes the object of a perpetual *simulation* of life. For this reason, however, a *form* of life is established in which “communication becomes its content”,^65^ and consequently a culture from which the “culture of life” is excluded, and, to put it in Nietzsche’s words, is sacrificed in view of the “great hunt”^66^ for the truth of the world:

The world in fact is a milieu of pure exteriority. Everything that finds within it the condition of its being is never offered as anything but external-being, a section of exteriority, a surface, a shoreline given over to our regard and upon which our regard glides indefinitely without being able to penetrate the inside of that which is concealed from view behind each new aspect, façade, and screen. For this being which is nothing other than exteriority has no interior, its law is becoming, the incessant outcropping of new faces and new planes and knowledge tracks this succession of lures, each of which presents itself to knowledge only to immediately cover over a being it does not possess and to redirect knowledge to another lure that does the same. No interior: nothing that is living, *that can speak in its proper name, in the name of what it* *experiences*, in the name of what it is. They can speak only in the name of “things,” only in the name of death: in advance of the world and its ecstatic disclosure what exhibits itself and ex-poses itself is only the always-at-hand, the always-outside—the object.^67^

This “ontological lie” about life situates life entirely within the horizon of the world and its objectivity thereby unveiling not life but, in fact, death. The “debacle of humanism in all its forms”^68^ is, according to Henry, thus nothing other than the necessary consequence of this barbarism that is internal to the modern spirit, which still calls for a defense of a genuine humanity precisely where it has already degraded humanity to a lifeless copy, that is, to an automaton.^69^

## The political and the temptations of transcendence

The barbarism of which Henry bespeaks is not only a crisis of culture in the sense of a self-destruction of those “practical laws” in accordance with which the self-augmentation of subjective potentialities is actualized. On the contrary, the logic of barbarism is also operative in the political domain. Nowhere is this exhibited more clearly than in the catastrophes of twentieth-century European politics. Henry examines this “form” of barbarism in his book, *Du communisme au capitalisme: Théorie d’une catastrophe*. I highlight here only its broad argument: fascism and totalitarianism succeed in leading the living to negate themselves in their own ineluctable lives in order to induce them to destroy others in violence:

Let’s say simply here that never can life as such be the origin of crime, that is, of an act turned against itself, unless it engages in the monstrous process of self-negation, which will be the distinctive trait of nihilism and fascism.^70^

Life’s ontological self-flight, as Henry has paradigmed it in *La barbarie*, is thus treated in light of its political manifestation, or what Henry calls the *hypostasis of the political*. This consists in the application of the political as a universal, on the basis of which each individual can both be certain of his own particularity and yet be damned to experience himself in his nullity vis-à-vis the universal. In other words, the *hypostasis of the political* is quite literally the exaltation of the political to the status of a real being. Thus viewed, the hypostasis of the political severs the connection to the immanent realization of life on the part of individuals along with their “pathic intersubjectivity,” and this severing subjects living beings and their invisible relations to each other to the judgment of the visible gaze of the state (which is epitomized in Hegel’s notion of the “light of the state” (*Staatslicht*):

The individual is nothing other than the occupant of a place delimited by the ensemble of processes that constitute the substance of society and being taken into account as such, as the affair of *civitas*, is what defines the political. The political is everything, the individual is nothing.^71^

The debasement of the individual defines, for Henry, the core of fascism, its attack on life as such, which is carried out not only directly but structurally.

Fascism always implies the debasement of the individual and, at the bottom of this will to debasement, there lies the will to deny it. This negation of the individual is what allows fascism to appear from the outset as a force of death—but who is the individual we are speaking of here? Under what aspect or in what part of his being must this be directed, attained and precisely negated so that we can speak of fascism? In what makes him a living being […], it is where the individual is an individual, where he is this singular individual, *in his life*, that fascism strikes. […] And it is in this that fascism is veritably a force of death.^72^

Fascism attacks life by seeking to throw life outside itself into the sphere of contingent objectivity whereas in reality life’s essence appears to itself, is given to itself and is manifest to itself by experiencing itself on the basis of its primordial arch-passivity. Fascism as Henry conceives it is an attack on the phenomenological essence of absolute life that constitutes every living. We are thus confronted with the groundlessness of the hypostasis of the political that is rendered visible in the institution of fascism, its phantasmatic incarnation in the “body of a people” (or even in a “class-consciousness,” etc.).

If a people has its reality in the individual, the negation of the individual is in truth a self-negation. The time of the political is the time of despair, the moment when life, or the individual, no longer believing in themselves and wishing to flee themselves, throw themselves outside of themselves and plunge into everything that could motivate such a flight and notably political existence, an existence devoted to the public cause (*chose*), to history, to society and its problems, and to everything that permits the individual to no longer live his own life and to forget himself.^73^

Yet, according to Henry, it is not only in the “hypostasis of the political” indicative of real existing socialism or fascism that the groundlessness of the political can appear. Doubtless there are totalitarian ideologies that can be understood to have instrumentalized man’s fear of the groundlessness of communal being-together, insofar as they project this fear onto the ontological inconsistency of the very individual that has been constructed by them.^74^ However, democracy, too, can fall prey to the hypostasis of the political. And so the collapse of totalitarian political systems should not detract, Henry so argues, from the ever-present reality that democracy can enlist state-sanctioned violence in its own interest to protect its boundaries against individuals.

According to Henry, the breakdown of democracy here is based not on contingent but indeed undoubtedly alarming events. This is also true in the case of the (seemingly unavoidable) self-legitimating “caprice” of the self-elected elite or the “incompetence” of the political representatives,^75^ which may ultimately be excluded as a sufficient reason for a definitive failure in the context of procedural democracies. For Henry, the decisive question concerns rather the fact that the democratic itself lacks grounding. What he interrogates is the foundation of democracy itself, namely, the freedom and equality of individuals, as they are determined by “human rights.” As he explains, human rights do not represent a *formal* principle. On the contrary, they make up the core of what one understands by “material democracy.” More precisely, this signifies that they should not be understood as “procedurally entitled attributes,” since on these premises they can, in principle, also be legitimately negated.^76^ From this reflection it follows that democracy ultimately cannot discover its principle within itself (with respect to human rights Levinas speaks of an “extra-territorial claim”): for “how can what lies before every decision result from this (sc. *extra*-*territorial claim*), how can it be founded on it?”^77^ Yet according to Henry this objection does not arise by chance, but rather follows, as he notes, from the concept of political *representation*.

In order to fulfill the democratic principle, political representation was invented, but political representation [as the substitution of individuals by delegates; MS] is the negation of the democratic principle.^78^

This line of inquiry opens up a field of enormous scope with respect to the political, for it implies the fundamental question of whether or not the basic values of democracy can be grounded, and if so, how, politically, ethically, or religiously. For Henry, the grounding of democracy can only be thought along ethical lines. The ethical, however, in relation to the essence of life—understood even as the unrefusable gift of a supreme power, i.e., as the “transcendental birth” of our “I can,” which is born out of the hyper-power of absolute life (*hyper*-*pouvoir de la vie*)—is synonymous with the religious. Religious and so-called “primitive” societies would have known, says Henry, about this supreme power: they lived with the fundamental life-knowledge that man *is not his own ground*; they lived this knowledge culturally, in art for instance, which was thus always religious. The “elimination” of the last religious societies in the West, the Christian society, which thinks the absolute as Life, was, according to Henry, the natural result following upon the new science that Galileo set into operation, “which places life out of play in favor of the material universe.”^79^ This Galilean reduction takes place especially where, as we saw, the democratic principle forms a fateful “alliance” with it.^80^ Because for a social organization that seeks to discover the principle of its organization in itself the ostensibly transcendent origin of the ethical–political must necessarily appear as extrinsic to the political domain itself, as if imposed from the outside, i.e., as an inadmissible exteriority (*extériorité irrecevable*). The democratic principle here defined props up a political system, as history makes evident, contradictory to the sacredness of life as it is expressed in Henry’s concept of the transcendental field of absolute life that gives birth to each of us as living ipseities. This transcendental field interior to the ego finds no place in a political domain funded by Galilean science, and indeed life can never do so. The reason it cannot do so is because the categories of freedom and equality so cherished by democracy are what Henry calls empty concepts. What Henry conveys by labeling them as *empty* is the fact that they fail to render intelligible the irrefusable gift of life by which each living self is co-original within a primordial “co-pathetic collectivity” that precedes any possible symbolic institution of the “social bond.” It is no surprise, then, that Henry claims that the consequences for our late modern societies are disastrous given that they possess an ethos preoccupied with modern science, individual freedom and exterior display. Henry formulates the consequences in what follows, even if polemically:

In fact, from the moment scientific knowledge is taken as the only true knowledge and the Galilean field of the material universe that it apprehends is taken as the sole reality, then what does not appear in such a field—absolute Life, which experiences itself outside the world, the Ipseity of this life that is its “experience of self,” any transcendental Self drawing its essence from this Ipseity […]. ‘The death of God,’ a dramatic leitmotif of modern thought attributed to some audacious philosophical breakthrough and parroted by our contemporaries, is just the declaration of intent of the modern mind and its flat positivism. But because this death of God destroys the interior possibility of man, since no man is possible who is not first a living Self and a ‘me’, it strikes at the very heart of man himself.^81^

From this insight into the true nature of the “death of God” as the distinguishing feature of the modern crisis of the subject ensues one of Henry’s further cardinal insights—namely, the insight into the loss of an originary ethos, an “ethos in accordance with life’s self-giving”^82^:

This is why, as soon as this condition fails to appear, the imprescriptible love of others also disappears. The other is now just another person, a person as people are […]. Forgetful of their veritable condition and the other’s veritable condition, they behave toward themselves and others as mere people. Then the whole edifying morality that wishes to found itself on the mere person, on the rights of man, discovers its emptiness, its prescriptions are flouted, and the world is given over to horror and sordid exploitation, to massacres and genocides. It is not by chance that in the twentieth century the disappearance of ‘religious’ morality has given rise not to a new morality, a ‘secular morality’, albeit a morality without any definite foundation, but to the downfall of any morality and to the terrifying and yet daily spectacle of that downfall.^83^

## Concluding remarks

What I have tried to underline in the preceding reflections is the unfathomed fact that the “immanent teleology of life”—its tendency as a force to incessantly surpass itself—at the moment when it runs dry and remains idle, or is reduced to “natural needs,” ultimately turns against itself and others, *qua* projections of the self-reproach of the alienated self. For such “empty subjectivity” remains, as Henry notes, an “avid subjectivity.”^84^ Even when life is reduced ideologically or scientifically it does not cease *living*. As “empty,” however, it is not only in an avid way that subjectivity escapes into ever new representations of life—in particular medially and procedurally generated representations—as well as into certain representations of “itself as an other”. In fact, subjectivity becomes ensnared in this self-flight and, at once, falls ever deeper into the fear of *being nothing*—a fear, with which subjectivity copes only by means of projecting itself precisely into these representations of an absent life—those transcendence-surrogates or “yet unexplored possibilities,” of which Nietzsche spoke.^85^ The “true transcendence,” “the immanence of life in every living being,”^86^ falls prey to a forgetting that, according to Henry, is in fact transcendental.^87^ Such a self-forgetting on the part of life, however, does not therefore merely represent a negation of life but in fact is an *expression of life itself*. When Henry speaks of a “second birth” that is supposed to set us free,^88^ we are nevertheless left to wonder if he has not hereby precisely broken up the “unshakable positivity”^89^ of life—i.e., the essential duplicity of appearing—and thereby effaced the self’s place in the world, giving the impression that the interior self is to be privileged at the expense of the self’s creative activity in the exterior world.

In transcendence, it is hence necessary to vindicate the life that apprehends itself in immanence, as nothing more but also nothing less than a “biotope.”^90^ Hence we must understand the self-forgetting of life as its transcendental condition of possibility. The last words of Christ, as they are transmitted through the Gospel of Matthew, also testify to this idea: “My God, my God, why have you forsaken me?” In them a kenotic truth of the world is expressed, for which we as living beings have to assume responsibility, as Jan Patocka argues in a commentary on Christ’s words:

There is nothing mystical here. I would say that it is in fact very simple. ‘Why have you forsaken me?’ – the answer lies in the question. What would have happened if you hadn’t forsaken me? Nothing. Something can happen only if you abandon me. He who sacrifices himself must go all the way to the end. He is ‘forsaken’ precisely so that there will be nothing there, nothing he can cling to. No thing – but this does not mean that this *nothing* does not in fact contain the whole, *das All*, in the words of the poet.^91^

## Footnotes

1 Henry (2003a, p. 275).

2 Ricœur (1998, p. 156).

3 Waldenfels (1990, pp. 349–50).

4 Henry (2008a, p. 37) construes the distinction between praxis and theory as follows: “I call praxis that knowledge [*savoir*] of life in which life constitutes at once the power (*pouvoir*) that knows and what is known, procuring for knowledge, in an exclusive manner, its content. What characterizes, as we have seen, such knowledge, in the absence of all ecstasis, is the fact that there is in it no possible relation to any ‘world’ whatsoever. On the contrary, I call theory that mode of knowledge that is defined by this relation.” Henry calls ‘scientific knowing’ that mode of knowing that corresponds to the object relation.

5 Janicaud (2000, pp. 73–4).

6 Henry (2003a, p. 101).

7 Janicaud (2000, pp. 73–4). The debates concerning the status of the “turn” among new French phenomenologists was unquestionably marked by a polemical tone. This polemic was not in the least conditioned by Janicaud’s own commentary and position of “minimalist phenomenology,” which is faithful to Husserl’s phenomenological rigor, and indeed so in this very book. Janicaud himself takes an intercessional position on the diverse replies to the “theological turn” in an elaborate and thorough way in *La phénomenologie éclatée* (Janicaud 1998). On this debate and Janicaud’s approach in particular, see Gondek and Tengelyi (2010).

8 Henry (2000, pp. 173–80, § 23).

9 I should note that I cannot take into consideration Michel Henry’s large work on Marx (*Henry* 1991), which is relevant here.

10 See Henry (2000, p. 54).

11 Cf. Henry (1973, pp. 47–133).

12 Henry (1973, p. 63).

13 A closer examination of this difficult concept can be found in Khosrokhavar (2001).

14 See Husserl (1966, § 39).

15 It should be noted that Hume already distinguishes between these two possible ways that the essence of selfhood can be grounded; see Tengelyi (2009, p. 401).

16 See Husserl (1999, p. 24): “*Every intellectual experience, indeed every experience whatsoever, can be made into an object of pure seeing and apprehension*, while it is occurring (*indem es vollzogen wird*). *And in this act of seeing it is an absolute givenness*.” As to Henry, this quote shows that the mode of intentional phenomenalization presupposes another mode of phenomenalization than seeing that takes place from a distance, namely *enactment* (*Vollzug*).

17 Fink (1966, p. 201).

18 Henry (2003b, p. 112).

19 This concept is elaborated by Depraz (2000).

20 See Tengelyi (2009, p. 402).

21 Henry (2008a, p. 169).

22 Henry (1973, p. 473).

23 Concerning this discussion on individualization, see Kühn (2006).

24 For this reason, Henry describes, to use the language employed in recent discussions about the status of consciousness and self-consciousness, a form of pre-reflexive self-consciousness referred to as a ‚sense of self’. Cf. Zahavi (2005, pp. 106, 116).

25 Tengelyi (2009, p. 403); cf. Henry (1973, pp. 616–617).

26 Hatem (2005, p. 254).

27 Henry (1973, p. 475).

28 Henry (2003a, p. 101).

29 Henry (2003a, pp. 115, 262).

30 On the self-generation of Life as the generation of the “First Living”, cf. Henry (2003a); on the generation of the living being as the “Son of God” or the “Son within the Son”, cf. Henry (ibid, chapters 6 and 7); on reciprocity, or, as the case may be, asymmetry as the decisive relations of this inner-Trinitarian process, which Henry utilizes to think the phenomenality of Life, cf. Rivera (2011). That Henry’s christological position is already adumbrated very early in *L’essence de la manifestation*, particularly in his interpretation of Eckhart, has been shown by Gondek and Tengelyi (2010, pp. 365 ff.); James G. Hart offers a critical reading, indebted to Husserls phenomenology, in Hart (1998).

31 This critique recurs in different variations. Cf. in addition to B. Waldenfels’ critique which we will cite later, Janicaud (2000).

32 Henry (2003a, p. 107).

33 On the structure of this antinomy, cf. Henry (2003a, pp. 198–200).

34 Henry (2003a, p. 107).

35 Henry (2003a, p. 107).

36 See Audi (2006, pp. 204–5), Gondek and Tengelyi (2010, pp. 130 ff.).

37 Henry (2003a, p. 200).

38 Henry (2000, pp. 207–208).

39 Henry (2000, p. 208).

40 Cf. Henry (2000, pp. 227–228).

41 Henry (2003a, p. 250). A study of such transcendental habitus and its affective phenomenological genesis in life is offered by Gély (2007).

42 If nothing else this implies a revolutionary reorientation of the so-called problematic of intersubjectivity, that no longer proceeds from the givenness of the ego, but rather from the aforementioned “condition of sonship” as a “preunifying essence” (Henry 2003a, p. 257). Henry carries this theme further in *Incarnation* in the context of a rereading of the concept of “the mystical body of Christ” (cf. Henry 2000, pp. 339–50); on Henry’s transformation of the problematic of intersubjectivity see Khosrokhavar (2002).

43 Husserl (1960, p. 26).

44 Henry (2003a, p. 107).

45 Henry (1973, p. 130).

46 Janicaud (2000, p. 78).

47 Waldenfels (1998, pp. 41–2).

48 Doesn’t the subjectivity, whose parousia is thought here, thus embody that “beautiful soul” whose “transparent purity” and lack of relation to the world that Hegel reproached Christianity for? (Hegel 1988, p. 236).

49 Henry (2003b, p. 117).

50 Cf. Hart (1999).

51 Husserl (1970, p. 299).

52 Henry (2003a, p. 260).

53 Cf., for example, Henry (2002).

54 Henry (1987).

55 The concept of “symbolic institution” goes back to M. Merleau-Ponty and is meant to designate “those events in experience which endow it with durable dimensions, in relation to which a whole series of other experiences will acquire meaning, will form an intelligible series or a history—or again those events which sediment in me a meaning, not just as a survival or residue, but as the invitation to sequel, the necessity of a future.” (Merleau-Ponty 1988, pp. 108–109). As Tengelyi (2007) argues, this concept can serve as a basic concept to develop an inherently phenomenological theory of culture.

56 Hart (1999, p. 260).

57 Husserl applies this concept, in addition, to the characterization of the genetic acquisition of egoic apperception structures (see Husserl 2001, p. 268), in the sense it takes on in the framework of his “generative analyses” in the ambit of his crisis writings where he describes the dynamic of generative meaning-formation (cf. Husserl 1993, p. 62).

58 For this reason, already in Husserl, in my view, we see the possibility that the meaning of such a history cannot be traced back to an unanimous *Urstiftung* or one identical *Endstiftung*, nor, moreover, can it ultimately be conceived teleologically or archeologically; a possibility however that Husserl could not pursue further.

59 Henry addresses this substitution in his book on Marx, in which he demonstrates that Marx’s “transcendental view” of society and economy serves to show that the living, or, as Marx says, “actual work” (*wirkliche Arbeit*) is the irreducible practical basis not only of the irreal representatives of the economy but also of the economic itself, that is, he reminds us that work itself is not the economic (see Henry 1991).

60 Henry (2008a, pp. 185–186).

61 Henry (2008a, p. 284).

62 Henry (2008a, p. 186).

63 Henry (2008a, p. 190).

64 Henry (2008a, p. 210).

65 Henry (2008a, p. 238).

66 Nietzsche (1998, p. 43, § 45).

67 Henry (2008a, p. 36).

68 Henry (2003a, p. 265).

69 In *I am the Truth*, Henry will take this up in a discussion of the Anti-Christ motif. For with Henry we can ask, what is the automaton other than the “statue of the Beast” (Revelation 13:14; cf. Henry 2003a, pp. 268–269), which—to put it in the words of Henry—stands for the belief that no transcendental Self is required to experience oneself, that, thus, no one has to be posited in his conditioning, nor does one require the mediation of Christ in the sense of the First-Living.

70 Henry (1990, p. 89).

71 Henry (2004a, p. 161).

72 Henry (1990. p. 94).

73 Henry (2004a, p. 162).

74 It is impossible to deal here with the respective thesis, i.e., that the success of totalitarianisms consists in the exploitation of the embodied subject’s ontological weakness; cf., however, the diverse accounts on this by Bergoffen (1990) and Rogozinski (1994–1995).

75 Henry (2004a, p. 162).

76 Cf. (Henry 2004a, p. 172).

77 Henry (2004a); cf. Henry (1990, p, 180). In political theory this thesis is known as the so-called “Böckenförde-Paradoxon:” “The free, secularized state lives off presuppositions that it cannot itself avouch.” (Böckenförde 1976, p. 60); Levinas also speaks on this: “These rights of man, that do not need to be conferred, are thus irrevocable and inalienable. Rights that, independently of any *conferral*, express the alterity or absolute of every person, the suspension of all *reference*: a violent tearing loose from the determining order of nature and the social structure in which each of us is obviously involved […]” (Levinas 1993, p. 117).

78 Henry (2004b, p. 172).

79 Henry (2004b, p. 170).

80 Henry (2004b, p. 175).

81 Henry (2003a, p. 265).

82 See on this conception of the ethos Kühn (1998).

83 Henry (2003a, p. 257).

84 Henry (1990, p. 222).

85 Nietzsche (1998, p. 43; § 45).

86 Henry (2000, p. 176).

87 On the complex topos of forgetting in Henry, see Steinbock (1999).

88 Henry (2003a, pp. 152–170 and esp. p. 270).

89 Henry (2008b, p. 97).

90 I take this concept from Longneaux (2001).

91 Patočka (1990, pp. 310–1).
